# A naturally occurring variation in the *BrMAM-3* gene is associated with aliphatic glucosinolate accumulation in *Brassica rapa* leaves

**DOI:** 10.1038/s41438-018-0074-6

**Published:** 2018-12-01

**Authors:** Jifang Zhang, Hui Wang, Zhiyuan Liu, Jianli Liang, Jian Wu, Feng Cheng, Shiyong Mei, Xiaowu Wang

**Affiliations:** 1 Institute of Vegetables and Flowers, Chinese Academy of Agricultural Sciences, 100081 Beijing, China; 2 Institute of Southern Economic Crops, Institute of Bast Fiber Crops, Chinese Academy of Agricultural Sciences, 410205 Changsha, China; 3 College of Horticulture, Qingdao Agricultural University, 266109 Qingdao, China

## Abstract

Glucosinolate profiles significantly vary among *Brassica rapa* genotypes. However, the molecular basis of these variations is largely unknown. In this study, we investigated a major quantitative trait locus (QTL) controlling aliphatic glucosinolate accumulation in *B. rapa* leaves. The QTL, which encompasses three tandem *MAM* genes and two *MYB* genes, was detected in two BC_2_DH populations. Among the five-candidate genes, only the expression level of *BrMAM-3* (Bra013007) was significantly correlated with the accumulation of aliphatic glucosinolates in *B. rapa* leaves. We identified a naturally occurring insertion within exon 1 of *BrMAM-3*, which is predicted to be a loss-of-function mutation, as confirmed by qRT-PCR. We determined that the loss of function was associated with the low glucosinolate content in *B. rapa* accessions. Furthermore, overexpressing the *BrMAM-3* gene resulted in an increase in total aliphatic glucosinolates in *Arabidopsis* transgenic lines. Our study provides insights into the molecular mechanism underlying the accumulation of aliphatic glucosinolates in *B. rapa* leaves, thereby facilitating in the manipulation of total aliphatic glucosinolate content in *Brassica* crops.

## Giving vegetables a nutritional upgrade

The identification of a gene controlling the production of a critical class of plant metabolites could enable researchers to engineer healthier crops. Vegetables such as cabbage and broccoli generate various glucosinolates as a defense mechanism against pests and disease. But these molecules also have nutritional and medicinal value, and researchers led by Xiaowu Wang at the Chinese Academy of Agricultural Sciences have homed in on a key step in their manufacturing process. Working with the model species *Brassica rapa*, Wang and colleagues learned that a gene called *BrMAM-3* performs the critical first step in synthesizing a major class of glucosinolates. Manipulation of this gene as well as those encoding the enzymes that subsequently shape the functional characteristics of the resulting glucosinolates could yield enhanced vegetables that are even healthier for consumers.

## Introduction

Glucosinolates are sulfur- and nitrogen-containing plant secondary metabolites that commonly occur in the order Brassicales, including important *Brassica* crops such as oilseed rape (*B. napus*), cabbage (*B. oleracea*, Capitata group), and broccoli (*B. oleracea*, Italica group), and the model plant *Arabidopsis thaliana*. Physical tissue or cell injury causes these amino acid-derived thioglycosides to co-occur with specific β-glucosidases called myrosinases and associated proteins, thereby generating an activated plant defense system known as the “mustard oil bomb”^1^. Glucosinolate derivatives such as isothiocyanates, thiocyanates, and nitriles have a wide range of biological functions, which include anti-carcinogenicity in humans^2–6^, anti-nutritional effects using seed meal in animals^7^, and insect pest repellent and fungal disease suppression^8,9^. Moreover, glucosinolates are responsible for the special flavors of *Brassica* vegetables such as *B. rapa* and *B. oleacrea*^10,11^. Due to their diverse roles in plant metabolism, animal nutrition, disease, and flavors, glucosinolates are a potential target for genetic manipulation and applications in crop improvement programs.

Glucosinolates are classified into aliphatic, aromatic, and indole glucosinolates, depending on their precursor amino acids^12,13^. Glucosinolate biosynthesis occurs in three independent stages: chain elongation of the precursor amino acid, formation of the core structure, and side-chain modifications. In *A. thaliana*, chain elongation is an iterative three-step process that operates predominantly on Met and results in up to six methylene groups, which contribute to the variations in glucosinolate structures. The committed step during Met side-chain elongation is catalyzed by methylthioalkylmalate synthase (MAM)^12,14–16^, which is derived from isopropylmalate synthase (IPMS) of Leu biosynthesis.^17^ Recent reports have confirmed that glucosinolate levels are controlled by at least six R2R3-MYB superfamily transcription factors^18–23^. In *A. thaliana*, the aliphatic glucosinolates genes are regulated by *AtMYB28*, *AtMYB29*, and *AtMYB76* genes^18–22^, whereas *AtMYB34*, *AtMYB51*, and *AtMYB122* control the formation of indole glucosinolates^23^. Additionally, MYC2, MYC3, and MYC4 regulate glucosinolate biosynthesis by directly interacting with glucosinolate-related MYBs^24^.

*Brassica* crops are of great economic importance to human beings because these are a rich source of beneficial health glucosinolates such as glucoraphanin and sulforaphane. Vegetable forms of *B. rapa* (Chinese cabbage, turnip, pakchoi, komatsuna, mizuna green, and rapini) are widely cultivated in many parts of the world^25^, and individual plants generally contain a limited number of major aliphatic glucosinolate profiles^10,26^. Glucosinolate content and profiles are highly variable and accession-specific in various *B. rapa* genotypes, in which the aliphatic (4C) 3-butenyl and the aliphatic (5C) 4-pentenyl glucosinolates are the predominant glucosinolates. To date, 102 putative genes of the glucosinolate biosynthesis pathway of *B. rapa* have been inferred by comparative genomic analyses^27^. The expression of seven MYB transcription factors in different organs of Chinese cabbage (*B. rapa* ssp. *Pekinensis*) has been investigated^28^. The expression profiles of *BrMYB28* and *BrMYB29* in stems differ from those in other organs^28^. In addition, three genes encoding AOP2 are differentially expressed in *B. rapa*^29^. However, more work is required to characterize the genes involved in glucosinolate biosynthesis in *B. rapa*.

The level and composition of aliphatic glucosinolates are under complex genetic control and are highly heritable^30^. Quantitative trait locus (QTL) analysis is a powerful method to study the genetics underpinning quantitative variations in glucosinolate profiles and to estimate the number of variable loci affecting a trait.^31^ QTL analysis of seed and leaf glucosinolates has been conducted in *A. thaliana*^9,14,32,33^, *B. oleracea*^34^, *B. napus*^35–37^, *B. juncea*^38,39^, as well as *B. rapa*^26^. In *Arabidopsis*, the epistatic interaction of two major genetic QTL controlling total aliphatic glucosinolates that map to the *GS-Elong* (*MAM1* and *MAM-L*) and *GS-AOP* loci regulate aliphatic glucosinolate accumulation^14^. In *B. rapa*, QTL analysis has identified 16 loci that control aliphatic glucosinolate accumulation, three loci that regulate total indolic glucosinolate concentration, and three loci that influence aromatic glucosinolate concentrations^26^. Although these reports have identified QTLs that control the variability of glucosinolate contents and profiles across *Brassica* species, its underlying molecular genetic mechanism remains unclear.

Genome polyploidization is an evolutionary process that fuels diversity in plant species^40^. Besides ancient whole-genome duplication events involving ancestral *Arabidopsis* and *Brassica* species^41^, *Brassica* crops underwent additional whole-genome triplication (WGT) events. These genome duplication events followed by gene losses during rediploidization resulted in highly complex relationships among the regulatory pathways of aliphatic glucosinolate biosynthesis in *Brassica* species compared to that in *Arabidopsis*. The *MAM* genes are often found as clusters of tandem arrays and differentiated in the *Arabidopsis* and *A. lyrata* genomes^9,12^. In *B. rapa*, seven *MAM* genes, comprising five syntenic and two non-syntenic, have been identified^27^; however, their function in relation to glucosinolate accumulation is poorly understood.

In our previous study, we analyzed the phylogenetic and syntenic relationships of *MAM* genes from 13 sequenced Brassicaceae species. Based on these analyses, we proposed that the syntenic loci of *MAM* genes underwent lineage-specific evolution routes and were driven by positive selection after the divergence from *Aethionema arabicum*^29^. Upon the divergence of the Brassica genus, *B. rapa* retained five syntenic *MAM* genes that were generated via WGT followed by biased gene loss. *BrMAM-1* and *BrMAM-2* are clustered in the medium-fractionated subgenome (MF1), whereas *BrMAM-3*, *BrMAM-4*, and *BrMAM-5* are clustered in the least-fractionated subgenome (LF). Furthermore, *BrMAM-3* and *BrMAM-1/2* are homologous genes. However, the contribution of these *MAM* genes in the observed variations in aliphatic glucosinolate accumulation of *B. rapa* remains unclear.

In this study, we investigated a major QTL locus controlling the accumulation of aliphatic glucosinolates in *B. rapa* leaves. The QTL locus includes a complex loci with three tandem *MAM* genes (*BrMAM-3*, *BrMAM-4*, and *BrMAM-5*) and a nearby loci with two *MYB* genes (*BrMYB28.1* and *BrMYB34.1*). All these genes are possibly involved in controlling aliphatic glucosinolate accumulation in *B. rapa*. The present study was conducted to clarify the gene(s) contributing to the significant QTLs involved in glucosinolate composition and accumulation in *B. rapa* leaves. Our study may facilitate the genetic engineering of plants to accumulate glucosinolates without compromising overall plant fitness.

## Results

### Expression of *BrMAM-3* is significantly associated with the accumulation of aliphatic glucosinolates

**Fig. 1:**
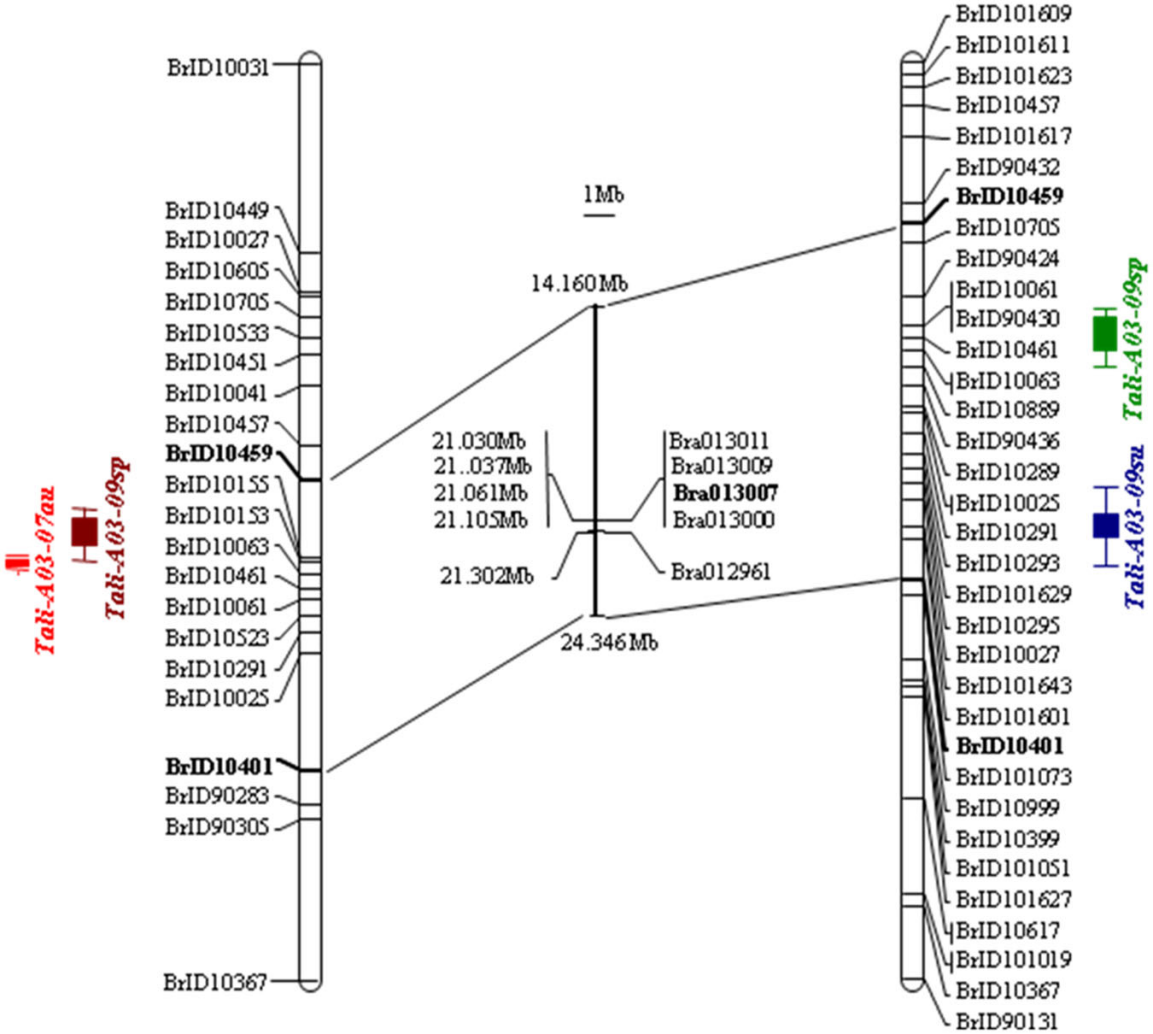
A major QTL locus on chromosome 3. A major QTL was mapped to a ~10.185-Mb genomic DNA region between markers BrID10459 and BrID10401 in RC_BC_2_DH (**a**) and YS_BC_2_DH (**b**) populations in autumn of 2007 and the spring of 2009, and in the spring of 2009 and the summer of 2009, respectively

A major QTL locus on chromosome 3 was consistently detected by MapQTL4 in the RC_BC_2_DH and YS_BC_2_DH populations, which explained a large proportion (31.0% and 53.0%, respectively, in autumn of 2007 and spring of 2009; 29.0% and 12.9%, respectively, in spring of 2009 and summer of 2009) of the observed phenotypic variations in total aliphatic glucosinolates (Fig. 1). There are eight genes (Supplementary Table S1, S2) in the major QTL region and five were considered as candidate genes, including three tandem *MAM* genes, namely, *BrMAM-3* (Bra013007), *BrMAM-4* (Bra013009), and *BrMAM-5*(Bra013011), and two *MYB* genes, namely, *BrMYB34* (Bra013000) and *BrMYB28* (Bra012961).

The association between the expression of candidate genes (Supplementary Table S3) and the glucosinolate profiles was analyzed in 52 double-haploid (DH) lines (Table 1). The profiles of eight glucosinolates, including three aliphatic,3-butenyl- (NAP), 4-pentenyl- (GBN), and 2-hydroxy-3-butenyl-glucosinolate (PRO), four indolic3-indolmethyl- (GBC), 1-methoxy-3-indoylmethyl- (NEO), 4-hydroxy-3-indoylmethyl- (4OH), and 4-methoxy-3-indoylmethyl-glucosinolate (4ME), and one aromatic glucosinolate, 2-phenylethyl-glucosinolate (NAS), were detected in all varieties. The total glucosinolate content in our collections ranged from 0.96 μmol g^−1^ dry weight (DW) to 46.02 μmol g^−1^ DW, with the aliphatic glucosinolates making up the highest ratio (75.5%, Table S1). NAP was the most abundant glucosinolate, with a mean content of about 3.98 μmol g^−1^ DW, representing 30.5% of the total glucosinolate content (Supplementary Table S4).

**Table 1:** Correlation analysis between expression of candidate genes and dominant glucosinolates in *Brassica rapa*

Genes	PRO	NAP	GBN	4OH	GBC	NAS	4ME	NEO	SUM
*BrMYB28.1* (Bra012961)	−0.06	0.25	0.12	0.06	0.10	0.04	0.28	0.03	0.25
*P* value	0.66	0.07	0.41	0.68	0.47	0.80	0.04	0.86	0.08
*BrMYB34.1* (Bra13000)	−0.10	0.42	0.21	0.46	0.06	0.13	−0.09	0.00	0.40
*P* value	0.46	0.00	0.14	0.00	0.66	0.37	0.52	1.00	0.00
*BrMAM-3* (Bra013007)	−0.13	0.70	0.07	0.43	0.01	0.03	−0.14	−0.06	0.57
*P* value	0.34	<0.0001	0.62	0.00	0.96	0.83	0.34	0.70	<0.0001
*BrMAM-4* (Bra013009)	−0.06	−0.14	0.24	−0.12	−0.01	0.07	−0.04	0.16	−0.03
*P* value	0.68	0.32	0.08	0.39	0.96	0.63	0.77	0.27	0.81
*BrMAM-5* (Bra013011)	−0.11	0.13	0.09	0.44	0.47	0.24	0.02	0.10	0.16
*P* value	0.43	0.36	0.53	0.00	0.00	0.08	0.88	0.48	0.27

Among the five-candidate genes, *BrMAM-3* (Bra013007) was significantly and positively associated with NAP (0.70) and the accumulation of total glucosinolates (0.57) (*P* < 0.001) as well as 4OH (0.43) (*P* < 0.01). *BrMYB34.1* (Bra13000) was associated with NAP (0.42), 4OH (0.46), and the accumulation of total glucosinolates (0.40) (*P* < 0.01). *BrMAM-5* (Bra013011) was associated with 4OH (0.44) and GBC (0.47) (*P* < 0.01). *BrMYB28.1* (Bra012961) and *BrMAM-4* (Bra013009) showed no correlation with glucosinolate accumulation. These results suggest that *BrMAM-3* plays an important role in controlling the accumulation of aliphatic glucosinolates. Our inference agrees with the fact that overexpression of the *AtMAM1* gene in *Brassica* spp. increases total aliphatic glucosinolate content^42^.

### Nucleotide polymorphisms of candidate genes situated within major QTLs

**Fig. 2:**
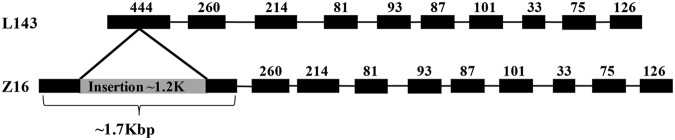
Gene structure of *BrMAM-3* in accessions L143 and Z16. Exons are shown in black blocks and numbers above the blocks indicate exon length. The gray block in exon one indicates the transposon insertion

We found that an insertion in *BrMAM-3* that was related to the observed variations in aliphatic glucosinolates. The B*rMAM-3* gene that was predicted consists of 11 exons, encoding 504 amino acids. The deduced protein sequence of the *BrMAM-3* gene showed 81.2% and 77.9% identity with AtMAM1 and AtMAM3, respectively. The *BrMAM-3* gene showed 100% identity with the L143 and Z16 accessions, except for one transposon insertion in exon 1. Furthermore, ten single-nucleotide substitutions (SNPs) across introns and exons were detected. Among the SNPs, three synonymous and one non-synonymous mutations were located in exons; the other SNPs were detected within introns, but did not affect the characteristics of amino acids. However, the *BrMAM-3* transcript containing the 1.2-kb transposon insertion fragment (Fig. 2) was predicted to be translated into a non-functional truncated protein of 101 amino acids that included a frame shift. We detected trace or no aliphatic glucosinolates in the Z16 accession, which harbors the *BrMAM-3* allele with the transposon insertion.

No correlation with aliphatic glucosinolates was observed for the variations in *BrMYB34.1*, *BrMYB28.1*, *BrMAM-4*, and *BrMAM-5*. The gene sequences showed 100% identity between L143 and Z16 accessions, except for several SNPs across introns and exons. For example, there were two SNPs involved in *BrMYB34.1*, three in *BrMYB28.1*, and five in *BrMAM-5* (Figure S1). These SNPs did not affect the characteristics of amino acids. Moreover, the *BrMAM-4* and *BrMAM-5* genes exhibited intact gene structures compared to *AtMAM*. The *BrMAM-4* gene contains eight exons, and the *BrMAM-5* gene has seven exons. These encode proteins with incomplete motifs, lacking two conserved motifs, respectively. The missing motifs belong to the PLN03228 (methylthioalkylmalate synthase) conserved domain, which may result in lower or inactive enzyme activity during methionine biosynthesis^29^. Therefore, the *BrMYB34.1*, *BrMYB28.1*, *BrMAM-4*, and *BrMAM-5* genes were not considered key candidate genes that control the accumulation of aliphatic glucosinolates in *B. rapa*. *BrMAM-1* and *BrMAM-2*, which are clustered as MF1, also had intact gene structures compared to *AtMAM*. *BrMAM-1* lost one exon and *BrMAM-2* encodes a protein that lacks two conserved motifs^29^. These two genes were not detected by QTL analysis, suggesting that although these have inherited the same *MAM* gene of *A. thaliana*, these did not inherit the key role in controlling aliphatic glucosinolates accumulation during rediploidization.

### Relationship between total aliphatic glucosinolates and nucleotide polymorphisms in *BrMAM-3*

**Fig. 3:**
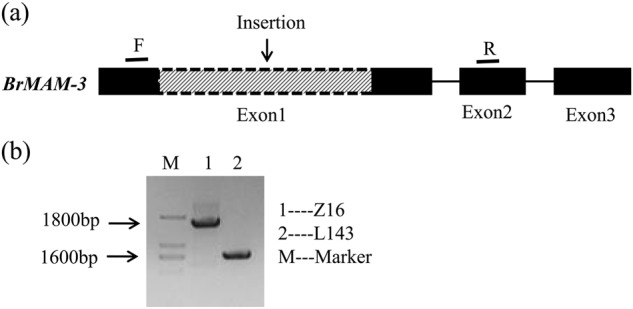
Schematic model of development of marker used to detect 1.2-kbp insertion mutation across exons 1 and 2 of *BrMAM-3*. **a** Insertion identified in accession Z16. Amplified region used to detect the insertion mutation encompassed part of exon 1, intron 1, and part of exon 2. F forward primer, R reverse primer. **b** Amplified fragments of *BrMAM-3* in the accessions L143 and Z16

**Fig. 4:**
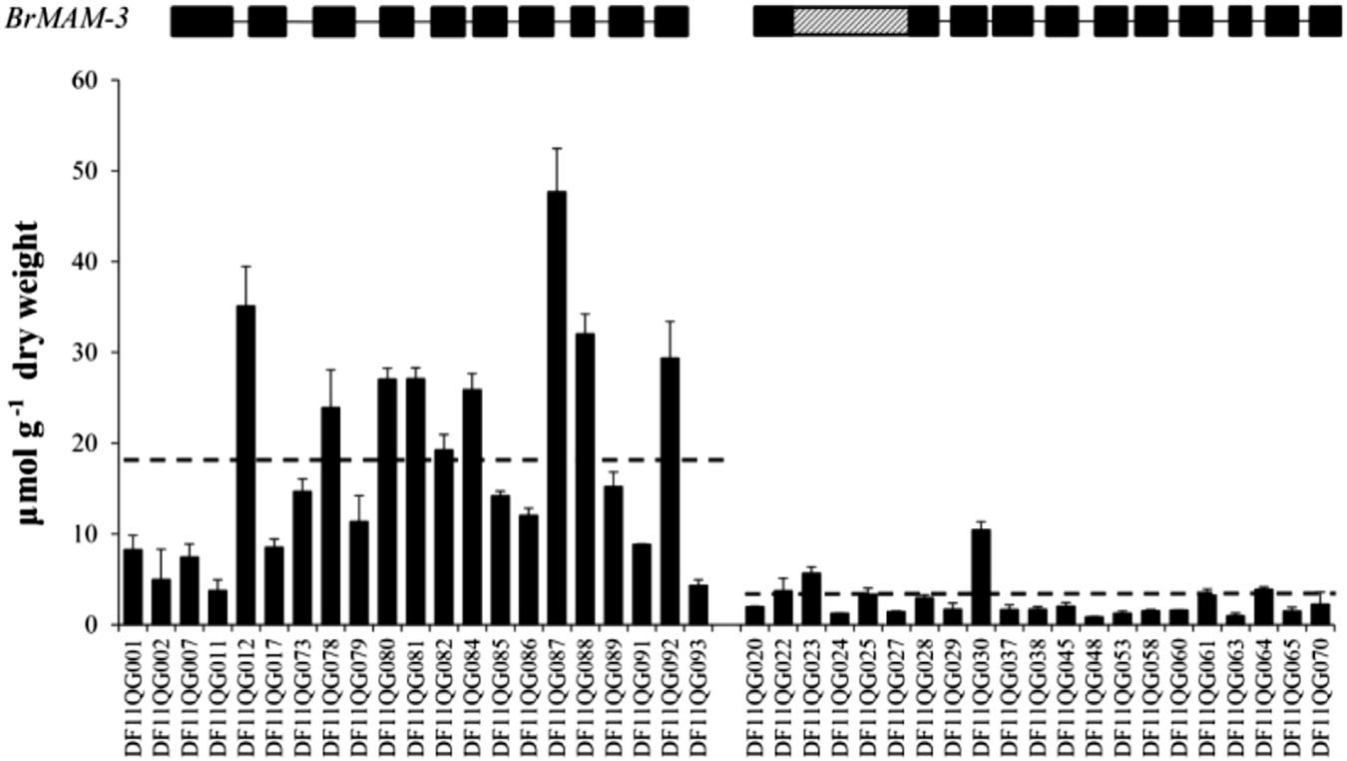
Amplified fragments of *BrMAM-3* and total aliphatic glucosinolates variations among germplasm collection of *B. rapa* leaves. Dashed lines indicate the average amount of total aliphatic glucosinolates

The insertion of *BrMAM-3* was used to develop a PCR-based marker across exons 1 and 2 and was validated in Z16 and L143 accessions (Fig. 3). The marker was used to analyze the association between total aliphatic glucosinolate accumulation and the *BrMAM-3* transposon-insertion alleles in a natural DH germplasm collection (Fig. 4). Correlation analysis showed that the transposon-insertion polymorphism is significantly associated with variations in the total aliphatic glucosinolate accumulation among the tested *B. rapa* accessions. Of the 42 screened DH lines, half of the accessions possessed the functional allele, whereas the others harbored transposon insertions (Fig. 4). The accessions with the functional alleles had a significantly higher amount of total aliphatic glucosinolates than those with insertion alleles. The average number of total aliphatic glucosinolates in DH lines with the functional *BrMAM-3* allele was about sixfold greater than those with the mutated insertion allele.

### BrMAM-3 is localized to the chloroplast

**Fig. 5:**
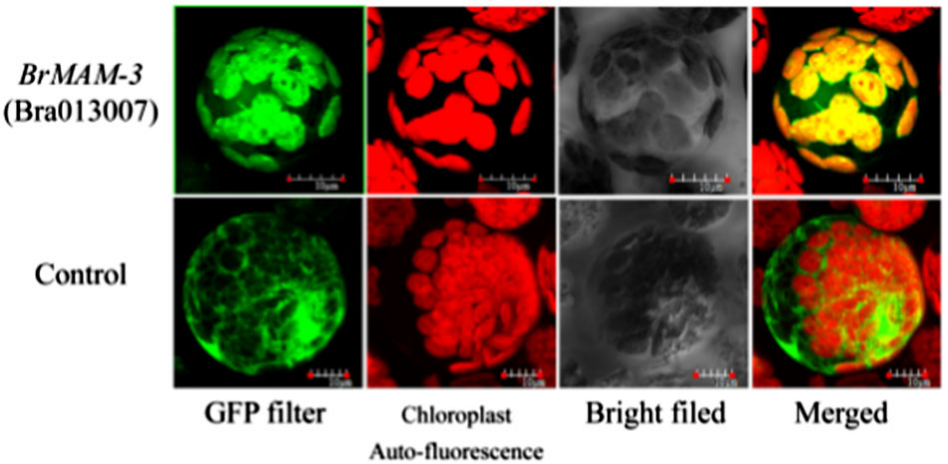
Subcellular localization of the BrMAM-3 protein in *Brassica rapa* protoplast. Images were taken in dark field for green fluorescence and chloroplast autofluorescence (in red color), whereas cellular morphology was assessed by imaging the cells in bright field. The bar indicates 10 μm

We investigated the subcellular localization of *BrMAM-3* to further analyze its characteristics. The Pro_CAMV35S_:BrMAM-3:GFP vectors were constructed and detected by monitoring the transient expression of GFP in *B. rapa* mesophyll protoplast cells (Fig. 5). The transiently transformed cells showed strong green fluorescence signals in the chloroplasts, demonstrating that BrMAM-3 protein is a predominantly chloroplast-localized protein, which agrees with that reported of *AtMAM3* in *Arabidopsis* (Col-0)^13,16^.

### BrMAM-3 encodes a functional protein that controls the accumulation of aliphatic glucosinolates

A complementation test was performed by creating transgenic plants overexpressing the *BrMAM-3* gene in the natural *Arabidopsis* mutant *Landsberg erecta* (Ler-0), in which the 5′ portion of *MAM1* is deleted^12^. Three independent homozygous lines of *BrMAM-3* were analyzed for total as well as individual glucosinolate fractions in 8-week-old rosette leaves.

**Fig. 6:**
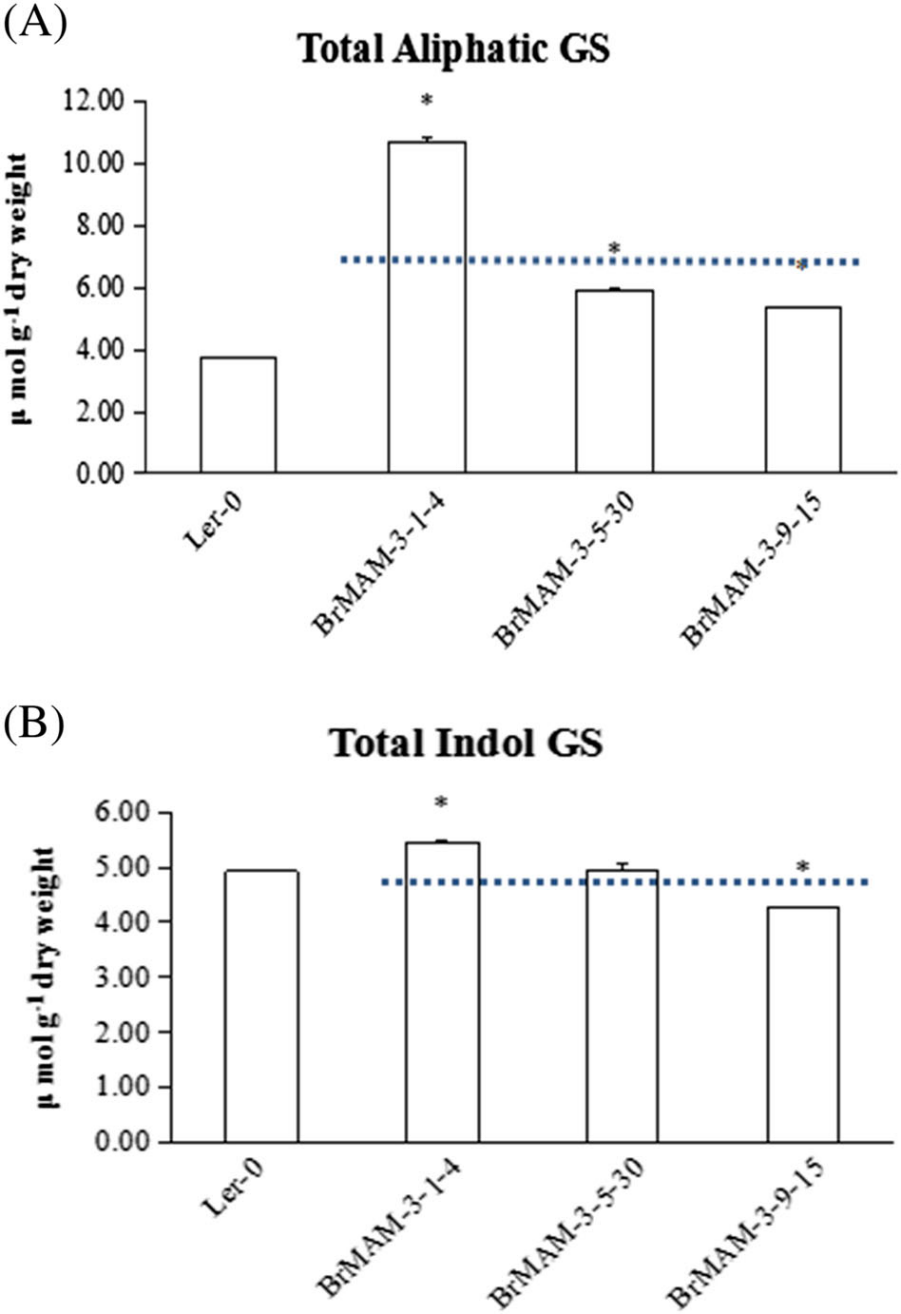
Functional complementation analysis of *BrMAM-3* gene in the *Arabidopsis* natural mutant Ler-0. The *BrMAM-3* gene is overexpressed in Ler-0, and the glucosinolate content and profile (in µnmol g^−1^ dry weight) was determined in 8-week-old rosette leaves. The individual graphs show the accumulation of **a** total aliphatic glucosinolates; **b** total indol glucosinolate. Three independent mutant-complemented lines of *BrMAM-3* gene were analyzed, and average foliar glucosinolates from 30 individual plants are represented along with their standard errors. Asterisks indicate significant differences in glucosinolate content compared with Ler-0 (*P* < 0.05, one-way ANOVA analysis with Duncan post hoc test). Dashed lines indicate the average amount of total aliphatic/indol glucosinolates

The functional complementation of the *BrMAM-3* gene elevated the accumulation of total aliphatic glucosinolate 1.4- to 2.9-fold compared to Ler-0 (Fig. 6a). However, the average amount of total indol glucosinolates of the complement lines did not exceed that of Ler-0 (Fig. 6b). Thus, the results of mutant complementation analysis in *A. thaliana* suggest that the *BrMAM-3* gene controls the levels of aliphatic glucosinolates.

### Expression patterns of *BrMAM-3* are associated with the accumulation of aliphatic glucosinolates in *B. rapa* leaves

**Fig. 7:**
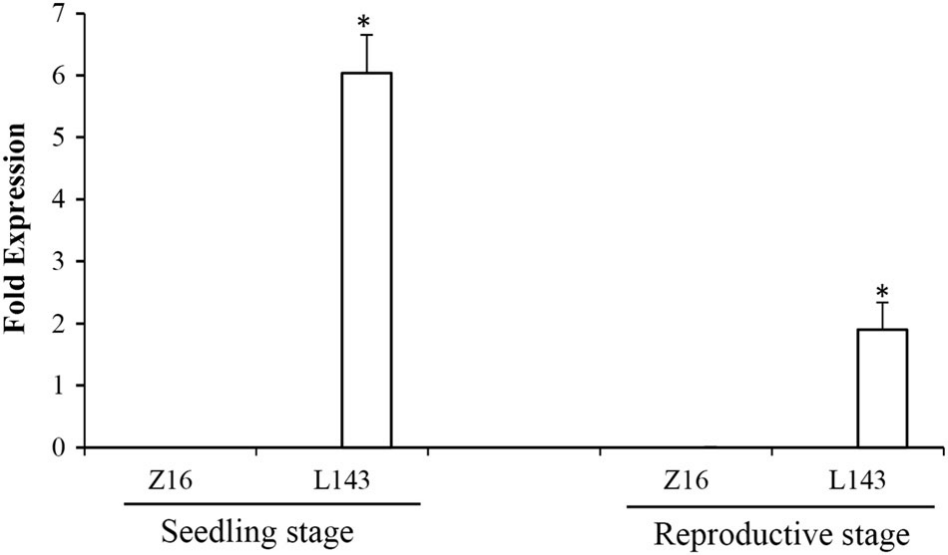
Expression profile of *BrMAM-3* gene at the seedling and reproductive stages in accessions Z16 and L143. Real-time quantitative PCR (qRT-PCR) was conducted, and the expression levels of *BrMAM-3* in L143 siliques are indicated. Error bars represent the standard deviation (SD) of three biological repeats. Asterisks indicate significant differences in the expression profile of the *BrMAM-3* gene compared to the Z16 accession (*P* < 0.05, determined by one-way ANOVA)

We measured *BrMAM-3* transcript expression in the accessions L143 and Z16 at the seedling and reproductive stages by qRT-PCR. The transcript levels of *BrMAM-3* were significantly higher in L143 than Z16 (Fig. 7). In Z16, undetectable or trace expression levels of *BrMAM-3* were observed in both the seedling and reproductive stages. In contrast, *BrMAM-3* was upregulated in glucosinolate-synthesizing tissues of L143 such as seedling leaves and mature leaves. The *BrMAM-3* expression profile in L143 coincided with the pattern of *AtMAM1* transcript accumulation, which showed maximum accumulation in expanding leaves followed by mature leaves.^16^

**Fig. 8:**
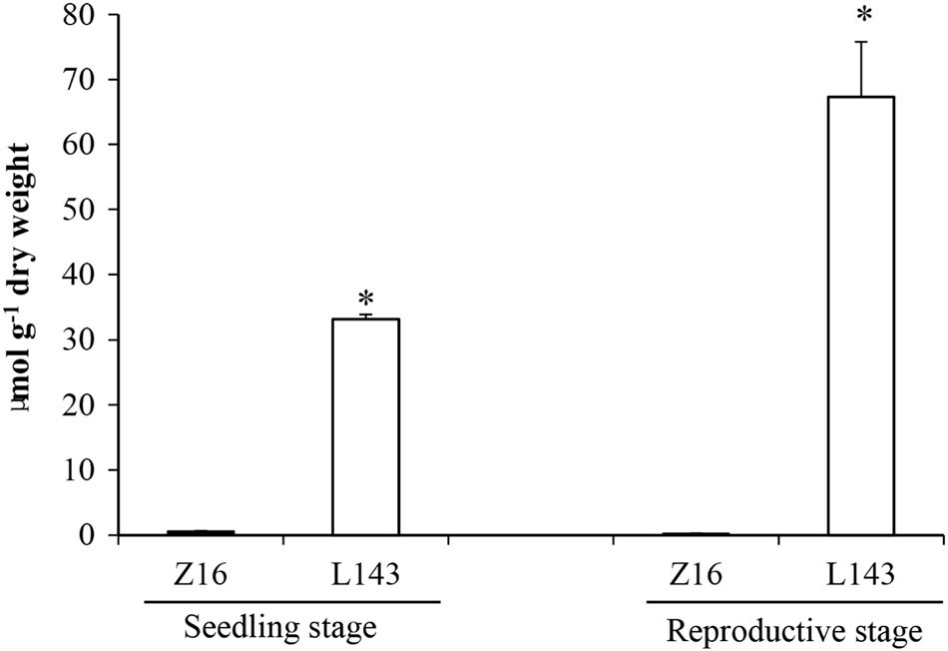
Total aliphatic glucosinolates accumulation (in µmol g^–1^ dry weight) in Z16 and L143 at the seedling and reproductive stages. Error bars represent the standard deviation (SD) of three biological repeats. Asterisks indicate significant differences in aliphatic glucosinolates content compared to the Z16 accession (*P* < 0.05, determined by one-way ANOVA)

To test whether the transcription of *BrMAM-3* gene contributes to the accumulation of total aliphatic glucosinolates in *B. rapa*, we analyzed the glucosinolate profiles of different developmental stages by high-performance liquid chromatography (HPLC). There were pronounced differences in the total aliphatic glucosinolate concentrations between Z16 and L143 both at the seedling and reproductive stages (Fig. 8). At the seedling stage, Z16 had trace amounts of total aliphatic glucosinolates in the leaves, whereas the expanding leaves of L143 depicted high aliphatic glucosinolate levels. These observations coincided with the expression pattern of *BrMAM-3*. In the reproductive stage, the total aliphatic glucosinolate in the leaves of L143 were higher than that of Z16. The total aliphatic glucosinolate accumulation in L143 at the seedling and reproductive stages was apparently inconsistent with the *BrMAM-3* expression patterns. Therefore, other *BrMAM* genes or the *R2R3-BrMYB* superfamily transcription factors may be involved in glucosinolate accumulation.

## Discussion

Aliphatic glucosinolates, secondary metabolites known to be involved in plant defense, make up the majority of the glucosinolate content in *A. thaliana* and *Brassica* spp. In *A. thaliana*, the amount of total aliphatic glucosinolates is regulated by three R2R3-MYB transcription factors (MYB28, MYB29, and MYB76), and their structural diversity arises from chain elongations of methionine and side-chain modifications. However, the molecular genetic mechanism controlling aliphatic glucosinolate accumulation in *B. rapa* is largely unknown. Our study implicates *BrMAM-3* in controlling aliphatic glucosinolate accumulation. This research will pave the way for the genetic manipulation of total aliphatic glucosinolate levels in *B. rapa*.

### Variations in *BrMAM-3* are related to reduced aliphatic glucosinolate levels in *B. rapa*

Apart from the artificially generated genotypes and high-throughput technologies, the analysis of naturally occurring genetic variations provides insights into the control of important plant processes. Relationships between naturally occurring variants and aliphatic glucosinolates have been reported in *A. thaliana*^43–45^ and *B. olerace*^46^, but are limited in *B. rapa* and other *Brassica* species. A previous report has shown that silencing of the *AtMAM* gene family in *B. napus* canola and *B. napus* rapeseed reduces the content of aliphatic and total glucosinolates^47^.

We analyzed the association between the expression and structure of five-candidate genes and glucosinolate content and profiles to study the genes involved in the differential aliphatic glucosinolate accumulation among *B. rapa* accessions. Our results showed that the two *MYB* genes (*BrMYB28.1* and *BrMYB34.1*) and two *MAM* genes (*BrMAM-4* and *BrMAM-5*) showed no significant correlation with aliphatic glucosinolates, *BrMAM-3* exhibited a significant positive correlation with aliphatic glucosinolates. Mutant complementation of *A. thaliana* showed that *BrMAM-3* increases the amount of total aliphatic glucosinolates. However, detailed expression analysis using qRT-PCR assays revealed that *BrMAM-3* has genotypic-specific expression partitioning in *B. rapa*. Moreover, a large insertion in exon 1 of *BrMAM-3* among the accessions with a trace amount of total aliphatic glucosinolate indicates that the loss function of *BrMAM-3* results in a reduction in total aliphatic glucosinolates in *B. rapa*. Meanwhile, the transposon insertion in the first exon of *BrMAM-3* could lead to a different transcriptional regulation of *BrMAM-3* between the L143 and Z16 accessions.

### Functional divergence could occur in the *MAM* of the major QTL

Based on the results of phylogenetic and syntenic analyses of sequenced Brassicaceae species, we propose a lineage-specific evolution pattern for syntenic *MAM* loci^48^. Upon divergence of the *Brassica* genus, *B*. *rapa* retained various copies of *MAM* genes, which were generated from WGT, followed by biased gene loss.

The three tandem *BrMAM* genes in the major QTL locus, with the same gene order and orientation, are located in the conserved syntenic region of the LF. *BrMAM-4* underwent a recent TD event to give rise to *BrMAM-5*^48^. The present study showed that *BrMAM-3* is responsible for the observed variations in aliphatic glucosinolates, indicating that *BrMAM-3* retained the function of the *MAMa* gene. Sequence analysis showed that *BrMAM-4* and *BrMAM-5* have intact gene structures compared to *AtMAM*, which lacks the methylthioalkylmalate synthase-conserved domain. Further investigation demonstrated that *BrMAM-4* is not associated with the accumulation of glucosinolates, indicating that *BrMAM-4* may have lost MAM enzyme activity. *BrMAM-5* is associated with 4OH and GBC but not aliphatic glucosinolates, suggesting that it acquired a new function (neofunctionalization) that is involved in indol glucosinoate biosynthesis.

*BrMAM-1* and *BrMAM-2* are located in the conserved syntenic region of the MF. *BrMAM-1* underwent a recent TD event to give rise *BrMAM-2*^48^. These two genes may have retained their catalytic activity for aliphatic glucosinoate accumulation, which requires validation in a future investigation.

### *BrMAM-3* is a candidate for engineering the high aliphatic glucosinolate trait in *B. rapa*

Glucosinolates have obtained the status of “model” secondary metabolites because their hydrolysis products exhibit different biological activities, e.g., as defense compounds and attractants. For humans, these compounds function as cancer-preventing agents, biopesticides, and flavor compounds. In the past decade, certain glucosinolates have been identified as potent cancer-preventing agents. Sulforaphane, the isothiocyanate derivative of glucoraphanin found in broccoli, has been the focus of many of these studies. These results aim to increase the sulforaphane content in broccoli and promote the health benefits of this vegetable. However, many Brassica species such as *B. rapa*, *B. napus*, and *B. juncea* harbor trace amounts of glucoraphanin, which is the precursor of sulforaphane^49^. RNA interference (RNAi) has been demonstrated to be an efficient method of silencing *GSL-ELONG*^47^ and *GSL-ALK* gene families^50^ to manipulate the beneficial glucosinolate profiles in *B. napus*. However, efforts in developing beneficial glucosinolate profiles in *B. rapa* are limited.

In *B. rapa*, the aliphatic glucosinolates are the predominant glucosinolates but vary among varieties in terms of content and glucosinolate profiles^10,51^. Our previous study identified three *BrAOP2* genes encoding the functional AOP2 that is involved in side-chain modifications of aliphatic glucosinolates^29^. Thus, it is possible to block *BrAOP2* genes to develop beneficial glucosinolates. In our current study, the accessions with functional *BrMAM-3* alleles had a significantly higher amount of total aliphatic glucosinolates than those with insertion alleles, demonstrating that the *BrMAM-3* gene plays an important role in controlling the accumulation of aliphatic glucosinolates in *B. rapa*. Thus, *BrMAM-3* could be utilized to improve the amount of total aliphatic glucosinolates in *B. rapa* accessions with low aliphatic glucosinolate content. Silencing of *BrAOP2* genes and overexpression of the functional *BrMAM-3* gene can be coupled to enrich the amount of beneficial glucosinolates in *B. rapa*.

Our findings provide functional evidence of expression partitioning of *BrMAM-3* gene in controlling aliphatic glucosinolate content. Our results suggest that the naturally occurring transposon insertion in exon 1 of *BrMAM-3* contributes largely to the observed variations in accumulation of total aliphatic glucosinolates in *B. rapa*. The information obtained in the current study may aid in manipulating the aliphatic glucosinolate content in *Brassica* crops using conventional breeding and/or transgenic approaches.

## Materials and methods

### Plant material

To characterize the association of glucosinolate profiles of *B. rapa* and the expression pattern of five-candidate genes, we performed transcriptome profile sequencing in 52 *B. rapa* accessions according to Cheng et al.^52^ (Supplementary Table S3). These accessions belong to 11 cultivar groups (Supplementary Table S5).

Two DH accessions Z16 and L143 were germinated and grown in a greenhouse at the Chinese Academy of Agricultural Sciences (Beijing, China) in the spring of 2011. Z16 is a Chinese cabbage accession with low glucosinolate content, whereas L143 is a yellow sarson accession with a high level of glucosinolates content. Leaf samples were collected from these plants for *BrMAM-3* gene expression and glucosinolates profiling. Three biological replicates of each sample were prepared under normal growth conditions (10 weeks after sowing). Different tissues were collected, flash frozen in liquid nitrogen, and kept at −80 °C until further use.

Forty-two *B. rapa* accessions (Supplementary Table S5) were used to screen for *BrMAM-3* sequence variations. The accessions were grown in a greenhouse in Beijing in the fall of 2011 to investigate aliphatic glucosinolate profiles. The temperature in the greenhouse was between 15 and 25 °C night/day.

*A. thaliana* ecotype *Landsberg erecta* (Ler) was used for functional complementation in vivo. Seeds of Ler-0 were plated on soil and cold-treated at 4 °C for 3 days in the dark. After stratification, seeds were transferred into a temperature-controlled growth chamber under short-day conditions (8 h light, 16 h dark) at 21–24 °C and 40% humidity.

For plants grown on Petri dishes, the seeds were surface sterilized with 75% (v/v) ethanol for 7 min and then washed thrice with sterile water. Seeds were sown on Murashige and Skoog (MS)-agar medium (one-half-strength MS salt, pH 5.8) and cold-treated at 4 °C for 2 days in the dark, then placed in a growth chamber (16 h of light at 22 °C and 8 h of darkness at 18 °C). Transgenic plants were selected by germination on half-strength MS medium containing kanamycin/hygromycin antibiotics and were subsequently treated as wild-type plants.

### Development of BC_2_DH populations, QTL mapping, and isolation and sequencing of candidate genes

The recurrent parents, L143 and L144 (with a high levels of glucosinolates), were used as the female and the donor as the male parent (Z16 accession) to generate the F_1_ generation. A single F_1_ plant (maternal) was backcrossed to the respective cultivars (paternal) to produce BC_1_F_1_ plants. Then, each BC_1_F_1_ plant was backcrossed a second time with the two cultivars. Two BC_2_DH populations designated as RC_BC_2_DH (derived from the cross L143) and YS_BC_2_DH (derived from the cross L144) were developed by another culture. A total of 250 BC_2_DH lines were obtained from the two BC_2_DH populations.

For the analysis of phenotypic measurement of aliphatic glucosinolates, 120 individual lines of each BC_2_DH populations were used and grown in the greenhouse in two different seasons. Linkage maps were constructed using JoinMap 4 and QTL analysis was performed with MapQTL4 (https://www.kyazma.nl/). QTL mapping was initially performed on transformed data with interval mapping (IM) followed by composite interval mapping, referred to as MQM mapping in MapQTL4. The significant cofactors for each MQM model were determined through an iterative automatic cofactor selection. The genome-wide logarithm of odds (LOD) significance threshold was obtained from permutation tests with 1000 replicates as implemented in MapQTL4.

To detect possible variations in the candidate genes involved on the major QTL locus in L143 and Z16 accessions, the candidate gene sequences were amplified with specific primers (Supplementary Table S6) from genomic DNA, and sequenced using an ABI3730XL analyzer and analyzed using ClustalX.

### Generation of transgenic plants

The coding sequences of *BrMAM-3* gene were isolated and amplified using L143 cDNA as template with the gene-specific primers, including restriction sites (*BrMAM-3*, forward primer with *Kpn*I restriction site: 5′-GGGGTACCATGGCTTCGTCACTTCTG-3′, reverse primer with *Xba*I restriction site: 5′-GCTCTAGATTATACCACAGAAGAAATC-3′), and ligated to a pEASY-T1 vector. Following sequence analysis, the pEASY-T1:*BrMAM-3* constructs were digested with *Kpn*I/*Xba*I and inserted into the pCambia 1300 vector driven by a CaMV35S promoter. The resulting construct was verified by DNA sequencing and subsequently transformed into *Agrobacterium tumefaciens* (strain GV3101). The binary vector pCambia 1300 containing a hygromycin resistance gene was utilized in the selection of transformed *Arabidopsis* lines. The floral infiltration method^53,54^ was used to transform natural mutant Ler-0 plants. Transgenic plants were selected by germination on half-strength MS medium containing 30 μg mL^−1^ hygromycin antibiotics and were subsequently treated as wild-type plants. The T2 generation derived from the selected plants was used to identify homozygous transformed lines. The T3 generation homozygous plants were subsequently employed in HPLC analysis.

### Construction of the Pro_CAMV35S_:BrMAM-3:GFP fusion plasmid and transformation of *B. rapa* mesophyll protoplast cells

To identify the subcellular localization of the *BrMAM-3* gene, Pro_CAMV35S_:BrMAM-3:GFP (green fluorescent protein) constructs were generated. The *BrMAM-3* coding sequences without stop codon were isolated and amplified from cDNA with the gene-specific primers including restriction sites (forward primer with an *Xba*I restriction site: 5′-TCTAGAATGGCTTCGTCACTTCTGAC-3′, reverse primer with *Kpn*I restriction site: 5′-TTGGTACC TACCACAGAAGAAATC-3′). The PCR-amplified *BrMAM-3* was inserted into the pSPYCE-35S/pUC-SPYCE vector by *Xba*I/*Kpn*I digestion and ligation. The resulting constructs were verified by DNA sequencing. The subcellular locations of the *BrMAM-3* genes were detected by monitoring the transient expression of GFP in *B. rapa* mesophyll protoplast cells^54^ on an OLYMPUS FV1200 Laser Confocal System. GFP fluorescence was imaged at an excitation wavelength of 488 nm, and the emission signal was detected between 495 and 530 nm for GFP and between 643 and 730 nm for chlorophyll autofluorescence.

### Glucosinolate extraction and HPLC analysis

The extraction and quantification of glucosinolates were performed by HPLC as previously described (Hehongju protocols, 2002). Lyophilized samples (0.2 g) were weighed in 15-mL plastic tubes and immersed in boiling methyl alcohol (5 mL) containing 100 µL benzyl glucosinolate as the internal standard. After 20 min of gentle shaking, samples were cooled at 4 °C and centrifuged at 3000 × *g* for 10 min. The supernatant (extract) was cleaned twice with 70% methyl alcohol. The extracts were loaded onto DEAE Sephadex A25 columns and desulfated overnight using purified sulfatase before HPLC. The column was then washed thrice with 0.5 mL deionized water, and the eluent that was filtered using a 0.45 μm membrane was used for HPLC analysis. Specific glucosinolates were identified by comparing retention times and UV absorption spectra with purified standards. Concentrations of individual glucosinolates were calculated as nmol mg^−1^ DW relative to the area of the internal standard peak using the respective response factors reported earlier^55^.

### Reverse transcriptase-mediated first-strand synthesis and real-time RT-PCR analysis

Total RNA was isolated from different organs of accessions L143 and Z16 using a total RNA extraction kit according to the manufacturer’s instructions (Sangon, http://www.sangon.com) and then treated with *DNa*se I (Sigma-Aldrich) to eliminate any DNA contamination. RNA purity was determined spectrophotometrically, and quality was determined by examining rRNA bands on 1% agarose gels. cDNAs were synthesized from ~2 μg of total RNA using TransScript First-Strand cDNA Synthesis SuperMix (Transgen, www.transgen.com.cn) with oligo(dT) as primer in a 20-μL reaction.

The specificity of the primers of *BrMAM-3* and *BrGAPDH* was verified by DNA sequencing after their PCR products were cloned into pEASY-T1 vectors. The efficiency of gene-specific *BrMAM-3* and *GAPDH* primer pairs was initially ascertained using a fourfold serial dilution of the L143 cDNA. A linear correlation coefficient (*R*^2^) of 0.95 and above was observed over a 64-fold dilution range, which reflected the high efficiency of each primer pair. Real-time quantitative PCR (RT-qPCR) was performed in a total volume of 15 µL, which included 2 µL of diluted cDNA, 0.5 µL of each primer (10 pM), and 7.5 µL of 2× SYBR Green Master Mixes (Thermo Fisher, USA) on an Eppendorf real-Time PCR system (Eppendorf, Germany), according to the kit manual. The RT-qPCR program was conducted at 95 °C for 2 min, followed by 40 cycles of 95 °C for 30 s and 60 °C for 60 s. The expression level of *BrGAPDH* was used as an internal control, and the expression of other genes was computed using the 2^–ΔΔCT^ method^56^. The primers used in this work are listed in Supplementary Table S7.

### Insertion marker analysis

The forward primer F (5′-CGTCCGTACAACAAGTCATCC-3′) in exon 1 and the reverse primer R (5′-AACTTAACACTACTCGCGGCC-3′) in exon 2 were designed to develop an insertion marker for *BrMAM-3*. PCR was performed under the following conditions: denaturation at 94 °C for 5 min, followed by 30 cycles of amplification (94 °C for 45 s, 55 °C for 45 s, and 72 °C for 1.5 min), and a final extension at 72 °C for 10 min. The PCR products were separated on a 1% agarose gel to determine the genotype of the insertion marker.

### Statistical analysis

Data were analyzed for statistical significance using one-way ANOVA with Duncan’s post hoc test using the SPSS software. A *P* value <0.05 was considered as significant.

## Electronic supplementary material

supplementary data.docx
