# Supplementary material for: A naturally occurring variation in the BrMAM-3 gene is associated with aliphatic glucosinolate accumulation in Brassica rapa leaves
Source: Hortic Res. 2018 Dec 1;5:69. doi: 10.1038/s41438-018-0074-6 (PMC6269504; doi:10.1038/s41438-018-0074-6)
Supplement: Supplementary file 1 — supplementary data.docx [file 41438_2018_74_MOESM1_ESM.docx]

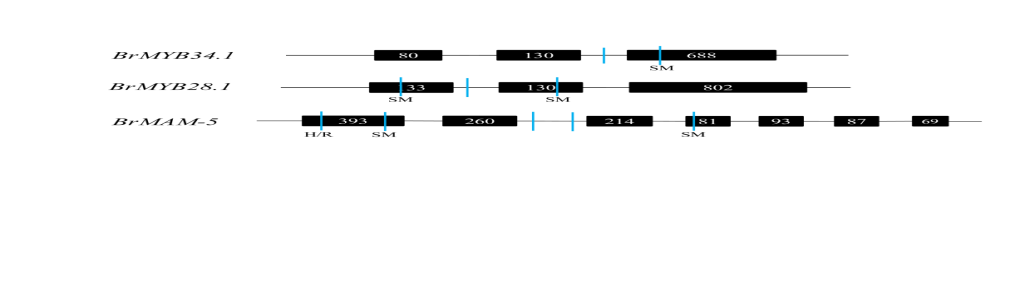


Figure S1. Allelic variations in *BrMYB34.1*, *BrMYB28.1* and *BrMAM-5* between accessions L143 and Z16. SM, synonymous mutation; H, histidine; R, arginine. Blue bars indicate SNP.

Table S1 Eight genes are involved in the QTL interval.

| Br Name | Br Gene | Annotation | GO ID |
| --- | --- | --- | --- |
| *BrIQD1-1* | Bra001299 | Calmodulin binding | GO:0019761 |
| *BrBCAT-4* | Bra001761 | Catalytic/ methionine-oxo-acid transaminase | GO:0010326 |
| *BrAPK1* | Bra013120 | ATP binding / adenylylsulfate kinase/ kinase/ transferase, transferring phosphorus-containing groups | GO:0010439 |
| *BrMAM-5* | Bra013011 | 2-isopropylmalate synthase/ methylthioalkylmalate synthase | GO:0010177 |
| *BrMAM-4* | Bra013009 | 2-isopropylmalate synthase/ methylthioalkylmalate synthase | GO:0010177 |
| *BrMAM-3* | Bra013007 | 2-isopropylmalate synthase/ methylthioalkylmalate synthase | GO:0010177 |
| *BrMYB34.1* | Bra013000 | DNA binding / kinase/ transcription activator/ transcription factor | GO:0005634 |
| *BrMYB28.1* | Bra012961 | DNA binding / transcription factor | GO:0010439 |

Table S2 *BCAT4-2* showed no correlation with the accumulation of glucosinolates.

|  | PRO | NAP | GBN | OH4 | GBC | NAS | ME4 | NEO | SUM |
| --- | --- | --- | --- | --- | --- | --- | --- | --- | --- |
| *BCAT4-2*(Bra001761) | -0.16 | 0.27 | 0.12 | 0.11 | 0.07 | 0.08 | -0.18 | 0.06 | 0.25 |
| p-value | 0.27 | 0.05 | 0.39 | 0.43 | 0.62 | 0.59 | 0.19 | 0.65 | 0.08 |
| *APK1*(Bra013120) | -0.05 | 0.21 | 0.11 | 0.21 | 0.28 | 0.39 | 0.07 | 0.09 | 0.27 |
| p-value | 0.75 | 0.13 | 0.42 | 0.13 | 0.04 | 0.00 | 0.64 | 0.53 | 0.05 |

Note: The assosication between expression of *BCAT4-2,* *APK1*and the glucosinolate profiles showed that the two genes had no correlation with the accumulation of aliphatic glucosinolates. Furthermore, we did not detect the expression of *IQD1-1* in all *B.rapa* accessions, indicating that *IQD1-1* is likely a pseudogene. So, *IQD1-1*, *BCAT4-2* and *APK1* are not considered as candidate genes.

Table S3 The expression of candidate genes in 52 accessions of *B. rapa*

| ID | Bra012961 (*BrMYB28.1*) | Bra013000 (*BrMYB34.1*) | Bra013007 (*BrMAM-3*) | Bra013009 (*BrMAM-4*) | Bra013011 (*BrMAM-5*) |
| --- | --- | --- | --- | --- | --- |
| DF001 | 0.7301 | 0.0000 | 0.7473 | 3.3485 | 1.9727 |
| DF002 | 17.2963 | 0.0000 | 11.0645 | 0.7631 | 14.2440 |
| DF003 | 11.4879 | 0.0000 | 0.0000 | 3.3506 | 0.0000 |
| DF005 | 5.6406 | 0.3623 | 0.1851 | 6.0044 | 4.0348 |
| DF006 | 0.2034 | 0.3052 | 0.0000 | 0.8449 | 2.0757 |
| DF007 | 4.8257 | 0.0000 | 1.5424 | 1.1594 | 3.0489 |
| DF011 | 2.6470 | 0.2957 | 35.1807 | 1.2791 | 3.7736 |
| DF012 | 7.3181 | 2.2600 | 59.8649 | 0.6622 | 6.4462 |
| DF013 | 4.1570 | 0.6975 | 0.0000 | 68.2566 | 6.3733 |
| DF015 | 4.2652 | 0.0000 | 39.1793 | 2.7457 | 3.3758 |
| DF016 | 0.9923 | 0.2052 | 0.0000 | 1.5137 | 2.1324 |
| DF017 | 2.2608 | 0.0000 | 19.7841 | 0.1023 | 6.7588 |
| DF019 | 2.0764 | 0.0000 | 1.2115 | 68.7197 | 12.8901 |
| DF020 | 6.4875 | 0.0000 | 0.6957 | 10.6746 | 4.2365 |
| DF021 | 7.3984 | 0.0000 | 0.0000 | 24.1092 | 2.4068 |
| DF022 | 8.9308 | 0.0000 | 0.0000 | 9.6698 | 0.9569 |
| DF023 | 0.9639 | 0.0000 | 0.0000 | 0.0205 | 4.0965 |
| DF024 | 3.1898 | 0.0000 | 0.0000 | 0.0000 | 1.5961 |
| DF025 | 1.7329 | 0.0000 | 0.0000 | 1.5275 | 2.5866 |
| DF026 | 0.4776 | 0.0000 | 0.0000 | 13.0110 | 0.4694 |
| DF027 | 1.8347 | 0.0000 | 0.0000 | 4.2712 | 0.4511 |
| DF028 | 10.8145 | 0.0000 | 0.0000 | 1.7294 | 0.9811 |
| DF029 | 12.7936 | 0.0000 | 0.0000 | 9.6679 | 0.7298 |
| DF030 | 0.6811 | 0.0000 | 0.0000 | 11.3038 | 0.9021 |
| DF032 | 0.0000 | 0.0000 | 0.0000 | 1.2146 | 1.8583 |
| DF033 | 0.0000 | 0.0000 | 0.0000 | 34.8545 | 0.7113 |
| DF034 | 18.4021 | 0.0000 | 0.7110 | 14.8218 | 3.9098 |
| DF035 | 3.0016 | 0.1965 | 0.7013 | 13.9653 | 3.4071 |
| DF036 | 5.7677 | 0.0996 | 0.6495 | 5.5072 | 3.6407 |
| DF037 | 5.4193 | 0.0000 | 0.0000 | 3.0655 | 3.2977 |
| DF038 | 10.9394 | 0.0000 | 0.0000 | 9.0209 | 2.5833 |
| DF039 | 8.8099 | 0.0000 | 0.0000 | 4.6269 | 1.2749 |
| DF040 | 3.5149 | 0.0000 | 0.0000 | 4.5691 | 1.1715 |
| DF041 | 4.1386 | 0.0000 | 0.0000 | 21.0614 | 1.4656 |
| DF042 | 11.2477 | 0.0000 | 0.0000 | 7.8251 | 0.8036 |
| DF043 | 3.1022 | 0.0000 | 0.0000 | 4.5641 | 1.1888 |
| DF045 | 0.0000 | 0.0000 | 0.0000 | 46.4492 | 1.0496 |
| DF046 | 3.0445 | 0.0000 | 0.0000 | 22.1352 | 0.2336 |
| DF047 | 3.3964 | 0.0000 | 0.0000 | 0.0000 | 0.9596 |
| DF048 | 5.3994 | 0.0000 | 0.0000 | 6.0231 | 0.8284 |
| DF053 | 2.5499 | 0.0000 | 0.0230 | 2.3306 | 1.2829 |
| DF055 | 13.6591 | 0.0000 | 0.0000 | 9.6654 | 0.5720 |
| DF056 | 8.1076 | 0.0000 | 0.0000 | 7.7692 | 1.9462 |
| DF058 | 5.0983 | 0.0000 | 0.0000 | 1.3864 | 1.5156 |
| DF060 | 7.3384 | 0.0000 | 0.0000 | 1.3760 | 1.3760 |
| DF061 | 1.2310 | 0.0000 | 0.0000 | 42.8892 | 1.3489 |
| DF063 | 3.2096 | 0.0000 | 0.0000 | 2.9803 | 0.9170 |
| DF064 | 5.0317 | 0.0000 | 0.0000 | 6.7117 | 2.0377 |
| DF065 | 9.4410 | 0.0000 | 0.0000 | 1.6418 | 1.1759 |
| DF066 | 0.4828 | 0.0000 | 0.2191 | 2.5886 | 2.0810 |
| DF068 | 2.5603 | 0.0000 | 0.0000 | 0.8281 | 0.8320 |
| DF070 | 2.2382 | 0.0000 | 0.0000 | 6.9648 | 0.8635 |
| DF071 | 6.1573 | 0.0000 | 0.0000 | 3.7329 | 1.7204 |
| DF073 | 3.3050 | 0.0000 | 0.5069 | 0.6387 | 1.2840 |
| DF078 | 5.2277 | 0.0000 | 1.0245 | 3.5719 | 1.4603 |
| DF079 | 0.9322 | 0.0000 | 0.0000 | 2.3823 | 1.2897 |
| DF080 | 16.0440 | 0.0000 | 14.6591 | 1.2397 | 2.4794 |
| DF081 | 15.2904 | 0.0000 | 21.2754 | 0.0232 | 0.9349 |
| DF082 | 2.0472 | 0.0000 | 0.0000 | 32.8730 | 1.9106 |
| DF083 | 0.3445 | 0.0000 | 0.3223 | 1.1727 | 1.0114 |
| DF084 | 3.2088 | 0.0000 | 0.5115 | 1.1002 | 1.9187 |
| DF085 | 1.8692 | 0.0000 | 1.8889 | 0.0000 | 1.1793 |
| DF086 | 16.1198 | 0.0000 | 0.0000 | 0.0000 | 0.9211 |
| DF087 | 12.8860 | 0.0000 | 1.0405 | 3.7430 | 0.8979 |
| DF088 | 2.9526 | 0.0000 | 0.0000 | 3.1987 | 4.6750 |
| DF089 | 6.2917 | 0.0000 | 0.0000 | 13.5745 | 0.0000 |
| DF091 | 9.8676 | 0.0000 | 0.0000 | 8.2700 | 1.6147 |
| DF092 | 10.8088 | 0.0000 | 7.1896 | 0.0146 | 2.8013 |
| DF093 | 18.4044 | 0.0000 | 3.0611 | 0.8890 | 0.7811 |

Note: expression values were estimated by TPM (tags per million reads)

Table S4 Mean and range (μmol·g^−1^ DW) for profiles and accumulation in 52 accessions of *B. rapa*

| Chemical class | Trivial name | Abbr |  | Percent  (%) | Mean  (μmol·g^−1^DW） | | Range  (μmol·g^−1^DW） |
| --- | --- | --- | --- | --- | --- | --- | --- |
| Systematic name |  |  |  |  |  |  |  |
| Total GS |  |  |  |  | 7.78 | 1.00-46.02 | |
| *Aliphatic* |  |  |  | 75.5 |  |  | |
| 3-Butenyl | Gluconapin | NAP |  | 30.5 | 3.98 | 0.02-29.46 | |
| 4-Pentenyl | Glucobrassicanapin | GBN |  | 25.0 | 1.94 | 0.02-14.40 | |
| 2-Hydroxy-3-butenyl | Progoitrin | PRO |  | 11.0 | 0.49 | 0.01-2.22 | |
|  |  |  |  |  |  |  | |
| *Indoyl* |  |  |  | 22.3 |  |  | |
| 3-Indolmethyl | Glucobrassicin | GBC |  | 6.7 | 0.30 | 0.01-1.15 | |
| 1-Methoxy-3-indoylmethyl | Neoglucobrassicin | NEO |  | 4.7 | 0.23 | 0.01-1.81 | |
| 4-Hydroxy-3-indoylmethyl | 4-Hydroxyglucobrassicin | 4OH |  | 0.4 | 0.02 | 0.00-0.12 | |
| 4-Methoxy-3-indoylmethyl | 4-Methoxyglucobrassicin | 4ME |  | 10.5 | 0.35 | 0.03-1.01 | |
|  |  |  |  |  |  |  | |
| *Aromatic* |  |  |  | 10.3 |  |  | |
| 2-Phenylethyl | Gluconasturtiin | NAS |  | 10.3 | 0.48 | 0.05-2.35 | |

Note: GS refers to glucosinolate.

Table S5 Accessions used for the analysis of *BrMAM-3* sequence variations

| **ID** | **Accessions** | **Subspecies** | **42** | | **52** |
| --- | --- | --- | --- | --- | --- |
| DF001 | V02B0645 | Pak Choi (ssp. *chinensis*) | ***** | | # |
| DF002 | Hei You Bai Cai | Pak Choi (ssp. *chinensis*) | ***** | | # |
| DF003 | Gao Jiao Shi Geng Bai | Pak Choi (ssp. *chinensis*) |  | | # |
| DF005 | Shi An You Cai | Pak Choi (ssp. *chinensis*) |  | | # |
| DF006 | Zhong He Qing Cai | Pak Choi (ssp. *chinensis*) |  | | # |
| DF007 | Shang Hai Qing | Pak Choi (ssp. *chinensis*) | ***** | |  |
| DF011 | Jing Sha Qing Jiang Bai Cai | Pak Choi (ssp. *chinensis*) | ***** | |  |
| DF012 | Zhao Cai Liao | Pak Choi (ssp. *chinensis*) | ***** | | # |
| DF013 | Si Yue Qi | Pak Choi (ssp. *chinensis*) |  | | # |
| DF015 | Wu Yue Man | Pak Choi (ssp. *chinensis*) |  | | # |
| DF016 | V02B0591 | Pak Choi (ssp. *chinensis*) |  | | # |
| DF017 | Hua Guan Qing Geng Bai Cai | Pak Choi (ssp. *chinensis*) | ***** | |  |
| DF019 | Lv Ling Tu Ta Cai | Wutacai (ssp. *narinosa*) |  | | # |
| DF020 | Zhong Ba Ye Tu Ta Cai | Wutacai (ssp. *narinosa*) | ***** | |  |
| DF021 | Z16 | Chinese cabbage (ssp. *pekinensis*) |  | | # |
| DF022 | Ju Long Kang Re Wang | Chinese cabbage (ssp. *pekinensis*) | ***** | |  |
| DF023 | Xian Feng Xia Yang | Chinese cabbage (ssp. *pekinensis*) | ***** | | # |
| DF024 | BrIVFhn P1 | Chinese cabbage (ssp. *pekinensis*) | ***** | | # |
| DF025 | BrIVFhn P2 | Chinese cabbage (ssp. *pekinensis*) | ***** | | # |
| DF026 | V02A0704 | Chinese cabbage (ssp. *pekinensis*) |  | | # |
| DF027 | Jian Chun | Chinese cabbage (ssp. *pekinensis*) | ***** | | # |
| DF028 | Shan Dong Si Hao | Chinese cabbage (ssp. *pekinensis*) | ***** | | # |
| DF029 | Lu Bai Er Hao | Chinese cabbage (ssp. *pekinensis*) | ***** | | # |
| DF030 | V02A0806 | Chinese cabbage (ssp. *pekinensis*) | ***** | |  |
| DF032 | 424-2-3 | Chinese cabbage (ssp. *pekinensis*) |  | | # |
| DF033 | Bei Jing 75 Hao | Chinese cabbage (ssp. *pekinensis*) |  | | # |
| DF034 | Huang Yang Bai | Chinese cabbage (ssp. *pekinensis*) |  | | # |
| DF035 | Luo Yang Da Bai Cai | Chinese cabbage (ssp. *pekinensis*) |  | | # |
| DF036 | 14 He Tao Wen Xiao Qing Kou | Chinese cabbage (ssp. *pekinensis*) |  | | # |
| DF037 | V02A1556 | Chinese cabbage (ssp. *pekinensis*) | ***** | |  |
| DF038 | Bei Jing Xin San Hao | Chinese cabbage (ssp. *pekinensis*) | ***** | | # |
| DF039 | Zao Shu 50 | Chinese cabbage (ssp. *pekinensis*) |  | | # |
| DF040 | Yu Xin 48 | Chinese cabbage (ssp. *pekinensis*) |  | | # |
| DF041 | Xin Feng 90 | Chinese cabbage (ssp. *pekinensis*) |  | | # |
| DF042 | L488-3 | Chinese cabbage (ssp. *pekinensis*) |  | | # |
| DF043 | 33--3 | Chinese cabbage (ssp. *pekinensis*) |  | | # |
| DF045 | Hua Bai Er | Chinese cabbage (ssp. *pekinensis*) | ***** | | # |
| DF046 | V02A1499 | Chinese cabbage (ssp. *pekinensis*) |  | | # |
| DF047 | V02A1351 | Chinese cabbage (ssp. *pekinensis*) |  | | # |
| DF048 | V02A1396 | Chinese cabbage (ssp. *pekinensis*) | ***** | | # |
| DF053 | Xia Kang 40 | Chinese cabbage (ssp. *pekinensis*) | ***** | | # |
| DF055 | hn53 | Chinese cabbage (ssp. *pekinensis*) |  | | # |
| DF056 | Kenshin | Chinese cabbage (ssp. *pekinensis*) |  | | # |
| DF058 | Zhu Long Cai | Chinese cabbage (ssp. *pekinensis*) | ***** | |  |
| DF060 | Qin Bai Er5-4-2 | Chinese cabbage (ssp. *pekinensis*) | ***** | |  |
| DF061 | Shi Te -1-3 | Chinese cabbage (ssp. *pekinensis*) | ***** | |  |
| DF063 | Yun Hong Zhong Jiang | Chinese cabbage (ssp. *pekinensis*) | ***** | |  |
| DF064 | Da Qing Ma Ye 939 | Chinese cabbage (ssp. *pekinensis*) | ***** | |  |
| DF065 | Si Ji Chun | Chinese cabbage (ssp. *pekinensis*) | ***** | | # |
| DF066 | Teng Xian Shao Zi Tou | Tai Cai (ssp. *parachinensis* Bailey) |  | | # |
| DF068 | F041464 | Tai Cai (ssp. *parachinensis* Bailey) |  | | # |
| DF070 | V02D0190 | Zi Caitai (ssp. *chinensis* var. *purpurea* Bailey) | ***** | | # |
| DF071 | Zi Caitai | Zi Caitai (ssp. *chinensis* var. *purpurea* Bailey) | | | # |
| DF073 | V02D0130 | Caixin (ssp. *parachinensis*) | | ***** | # |
| DF078 | 0134 | Yellow Sarson (ssp. tricolaris) | | ***** | # |
| DF079 | CGN17278 | Caixin (ssp. *parachinensis*) | | ***** | # |
| DF080 | R-o-18 | Yellow Sarson (ssp. *tricolaris)* | | ***** |  |
| DF081 | L144 | Yellow Sarson (ssp. *tricolaris)* | | ***** | # |
| DF082 | L41 | Komatsuna (ssp.perviridis) | | ***** | # |
| DF083 | CGN17281 | Komatsuna (ssp.perviridis) | |  | # |
| DF084 | Mizuna | Mizuna (ssp. *nipposinica*) | | ***** |  |
| DF085 | Mizuna | Mizuna (ssp. *nipposinica*) | | ***** | # |
| DF086 | CGN15199 | Turnip rape (ssp. *oleifera*) | | ***** | # |
| DF087 | CGN15220 | Turnip rape (ssp. *oleifera*) | | ***** | # |
| DF088 | CGN15201 | Turnip rape (ssp. *oleifera*) | | ***** | # |
| DF089 | CGN06721 | Turnip rape (ssp. *oleifera*) | | ***** | # |
| DF091 | SynBr01 | Chinese cabbage (ssp. *pekinensis*) | | ***** |  |
| DF092 | SynBr02 | Rapid cycling | | ***** |  |
| DF093 | SynBr03 | Chinese cabbage (ssp. *pekinensis*) | | ***** |  |

Note:# Accessions used in the association analysis. * Accessions used in the analysis of *BrMAM-3* sequence variations.

Table S6 Primer sequences of candidate genes involved in the major QTL locus

| **Gene ID** | **引物序列( 5’-3’)** |
| --- | --- |
| *BrMYB34.1* F | CTTCCTTTCGTAGGTCTCTAG |
| *BrMYB34.1* R | TATTATTCCCGCCTCGTGGCC |
| *BrMYB28.1* F | GTGGGTAAGACCCAAGAGTG |
| *BrMYB28.1* R | GTAATGAAGACTCTCATATGAGGG |
| *BrMAM-5* F  *BrMAM-5* R | CTCCAAATCGAAATCTCTCG  CAACGCTTCCATGGAACCC |

Table S7 Primer sequences of genes used in RT-qPCR analysis

| **Gene ID** | **Primer sequence( 5’-3’)** |
| --- | --- |
| *BrMAM-3* F | GGCAGGTCGGAGAAGGAGT |
| *BrMAM-3* R | CACATACACCGGCAATTGTG |
| *BrGAPDH*(Bra016729) F | CCACTTGCCAAGGTTATCAACGAC |
| *BrGAPDH*(Bra016729) R | CAACTGAAACATCAACGGTGGG |
